# Supplementary figures and images for: Prognostic Thresholds of Mitotic Count and Ki-67 Labeling Index for Recurrence and Survival in Lung Atypical Carcinoids
Source: Cancers (Basel). 2024 Jan 24;16(3):502. doi: 10.3390/cancers16030502 (PMC10854613; doi:10.3390/cancers16030502)

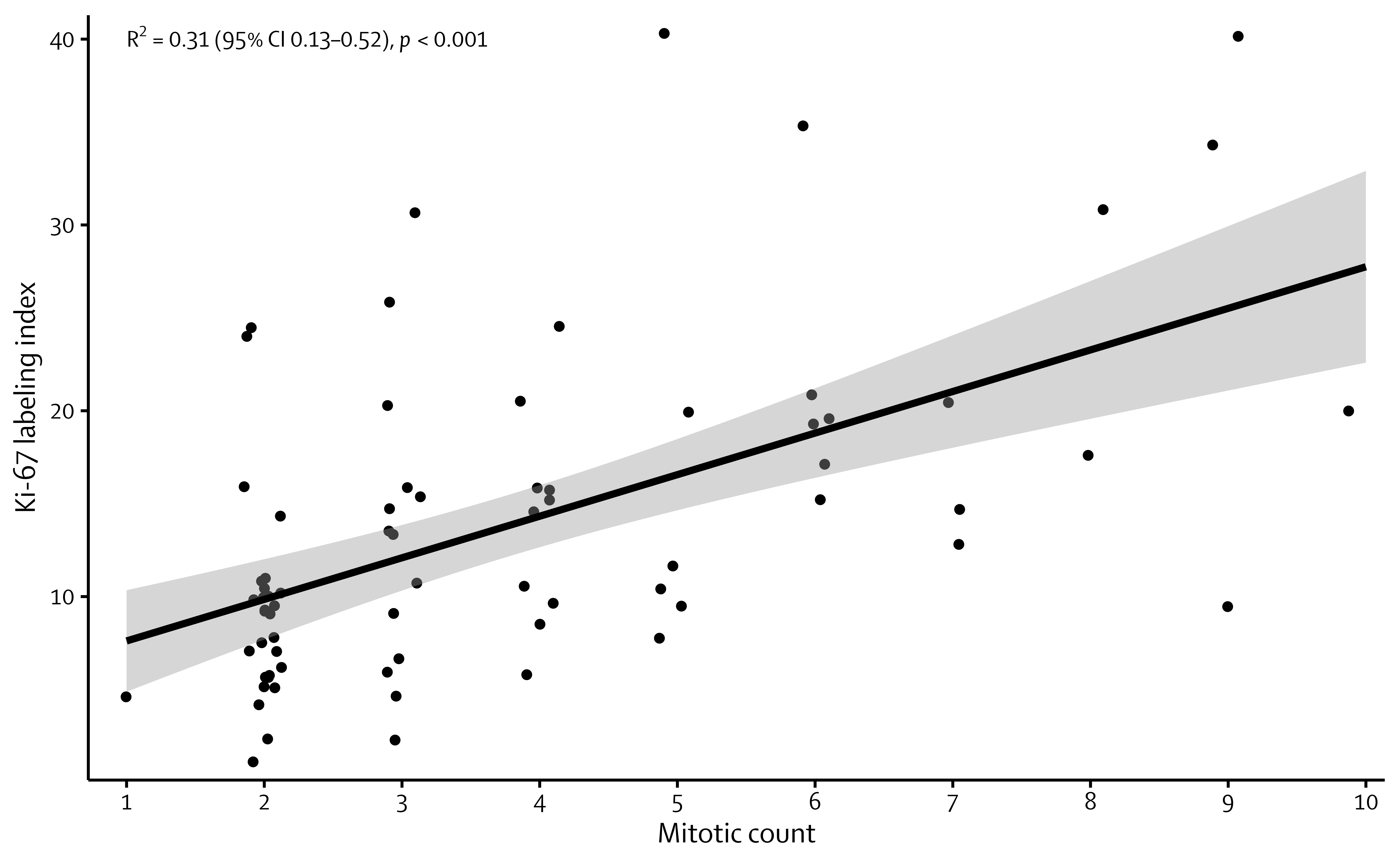

Supplement: Supplementary file 1 [file cancers-16-00502-s001.zip › Figure S1.png]
